# Cardiac Alterations on 3T MRI in Young Adults With Sedentary Lifestyle-Related Risk Factors

**DOI:** 10.3389/fcvm.2022.840790

**Published:** 2022-02-22

**Authors:** Gert J. H. Snel, Maaike van den Boomen, Katia Hurtado-Ortiz, Riemer H. J. A. Slart, Vincent M. van Deursen, Christopher T. Nguyen, David E. Sosnovik, Rudi A. J. O. Dierckx, Birgitta K. Velthuis, Ronald J. H. Borra, Niek H. J. Prakken

**Affiliations:** ^1^Department of Radiology, Medical Imaging Center, University Medical Center Groningen, University of Groningen, Groningen, Netherlands; ^2^Department of Radiology, Athinoula A. Martinos Center for Biomedical Imaging, Massachusetts General Hospital and Harvard Medical School, Boston, MA, United States; ^3^Cardiovascular Research Center, Massachusetts General Hospital and Harvard Medical School, Boston, MA, United States; ^4^Faculty of Medicine, National Autonomous University of Mexico (UNAM), Ciudad Universitaria, Mexico City, Mexico; ^5^Department of Nuclear Medicine and Molecular Imaging, Medical Imaging Center, University Medical Center Groningen, University of Groningen, Groningen, Netherlands; ^6^Department of Biomedical Photonic Imaging, Faculty of Science and Technology, University of Twente, Enschede, Netherlands; ^7^Department of Cardiology, University Medical Center Groningen, University of Groningen, Groningen, Netherlands; ^8^Harvard-MIT Division of Health Sciences and Technology, Cambridge, MA, United States; ^9^Department of Radiology, University Medical Center Utrecht, University of Utrecht, Utrecht, Netherlands

**Keywords:** young adults, cardiovascular magnetic resonance imaging, overweight, hypertension, type 2 diabetes, cardiac function, myocardial tissue characterization

## Abstract

**Background:**

Young adult populations with the sedentary lifestyle-related risk factors overweight, hypertension, and type 2 diabetes (T2D) are growing, and associated cardiac alterations could overlap early findings in non-ischemic cardiomyopathy on cardiovascular MRI. We aimed to investigate cardiac morphology, function, and tissue characteristics for these cardiovascular risk factors.

**Methods:**

Non-athletic non-smoking asymptomatic adults aged 18–45 years were prospectively recruited and underwent 3Tesla cardiac MRI. Multivariate linear regression was performed to investigate independent associations of risk factor-related parameters with cardiac MRI values.

**Results:**

We included 311 adults (age, 32 ± 7 years; men, 49%). Of them, 220 subjects had one or multiple risk factors, while 91 subjects were free of risk factors. For overweight, increased body mass index (per SD = 5.3 kg/m^2^) was associated with increased left ventricular (LV) mass (+7.3 g), biventricular higher end-diastolic (LV, +8.6 ml), and stroke volumes (SV; +5.0 ml), higher native T_1_ (+7.3 ms), and lower extracellular volume (ECV, −0.38%), whereas the higher waist-hip ratio was associated with lower biventricular volumes. Regarding hypertension, increased systolic blood pressure (per SD = 14 mmHg) was associated with increased LV mass (+6.9 g), higher LV ejection fraction (EF; +1.0%), and lower ECV (−0.48%), whereas increased diastolic blood pressure was associated with lower LV EF. In T2D, increased HbA1c (per SD = 9.0 mmol/mol) was associated with increased LV mass (+2.2 g), higher right ventricular end-diastolic volume (+3.2 ml), and higher ECV (+0.27%). Increased heart rate was linked with decreased LV mass, lower biventricular volumes, and lower T_2_ values.

**Conclusions:**

Young asymptomatic adults with overweight, hypertension, and T2D show subclinical alterations in cardiac morphology, function, and tissue characteristics. These alterations should be considered in cardiac MRI-based clinical decision making.

## Introduction

Increased western diet consumption combined with a sedentary lifestyle drives the global prevalence of being overweight and having obesity (1). While previously mostly seen in aging populations, today younger populations are more frequently affected (2). Consequently, the onset of both hypertension and type 2 diabetes (T2D) is also shifting to younger age groups (3, 4), subsequently increasing cardiovascular disease prevalence (5).

Non-ischemic cardiomyopathy is the most frequent cause of sudden cardiac death in the young, whereas the prevalence of ischemic cardiomyopathy increases with age (6). Young adults with non-specific cardiovascular symptoms are therefore often referred for MRI to rule out non-ischemic cardiomyopathy (7). Pathological cardiac MRI findings include left ventricular hypertrophy (LVH), chamber dilatation, and decreased ejection fraction (EF) (8). Early changes in cardiomyopathy are subtle and could overlap comparable alterations in overweight, hypertension, and T2D (9). Large cohort studies in mature adults (mean age, 55–62 years old) show overweight to be independently associated with increased LV mass and cardiac volumes, hypertension with increased LV mass, and T2D with smaller volumes (10–13). As included populations were asymptomatic and free of cardiovascular disease, the aforementioned alterations can be considered risk factor-related cardiac adaptation. To our best knowledge, the impact of these risk factors in younger adults has not been demonstrated yet.

Quantitative myocardial tissue characterization has an increasingly complementary role in the diagnosis of non-ischemic cardiomyopathies (14). For example, cardiomyopathies featuring myocardial fibrosis or edema show respectively prolonged native T_1_ and T_2_ mapping values (15, 16), and in some cases, also increased extracellular volume (ECV) (17). In high-risk populations, these mapping values could be different, which should be taken into account to avoid misdiagnosis. In athletes, for example, native T_1_ and T_2_ mapping and ECV have already been described to be discriminative for the athlete's heart and cardiomyopathy (14, 18). Previous studies in mature hypertensive populations have reported higher native T_1_ values and ECV in presence of LVH (19), however, as these increases were smaller than seen in patients with hypertrophic cardiomyopathy (HCM), these markers may serve as potential discriminators (20). In mature patients with T2D, ECV was mainly higher without clear changes in native T_1_ values (21). In both younger and older adult obese populations, no differences were reported in native T_1_ values and ECV (22, 23). T_2_ mapping values have only been reported in mature hypertensive and T2D subjects, both show higher values than in healthy controls (24, 25).

The aim was to investigate the impact and possible combined effects of overweight, hypertension, and T2D on cardiac morphology, function, and tissue characteristics in an asymptomatic young adult population.

## Methods

### Study Population

This prospective cross-sectional single center study was approved by the local Medical Ethics Review Committee, in accordance with the declaration of Helsinki, and all subjects signed informed consent prior to participation. We recruited individuals aged 18–45 years old with at least one cardiovascular risk factor: overweight, hypertension, or T2D. Overweight was defined as a body mass index (BMI) ≥ 25 kg/m^2^, hypertension as under pharmacological treatment or three consecutive blood pressure measurements ≥ 140/90 mmHg, and T2D as under pharmacological treatment or hemoglobin A1c (HbA1c) ≥ 48 mmol/mol. Healthy individuals without cardiovascular risk factors were additionally included as controls. All subjects were recruited using public advertisements and scanned between August 2017 and July 2019 strictly for study purposes without a clinical indication. Exclusion criteria for participation were cardiac history, cardiac symptoms, and cardiovascular comorbidities. Current smoking and exercising (>3 h/wk) were also exclusion criteria to minimize the impact of other covariates on the heart (10, 26). Body weight, blood pressure, and hip and waist circumference were measured. Body weight was measured while wearing only MRI-safe clothes and rounded down to correct for this. Blood pressure was measured in a sitting position after resting for at least 5 min (27). Blood samples were obtained shortly before the MRI examination to assess HbA1c, glucose, and hematocrit.

### MRI Data Acquisition

All study participants underwent cardiac MRI on a 3Tesla scanner (MAGNETOM Prisma, Siemens Healthineers, Erlangen, Germany) equipped with a 60-channel phased-array coil. All examinations were performed by experienced operators using breath-holds and electrocardiographic gating.

Steady-state free precession (SSFP) sequences were used to acquire short-axis cines covering the entire heart with a slice thickness of 6 mm, an interslice gap of 4 mm, and 25 phases per cardiac cycle (28). Long-axis cines were acquired with the same settings and included a single slice in 4-chamber view, and in 2-chamber view and outflow tract of the left and right ventricle. Detailed imaging parameters are provided in Supplementary Table S1.

T_1_ mapping was performed using a Modified Look-Locker Inversion Recovery 5(3)3 sequence on a basal, midventricular, and apical 8 mm short-axis slice (14). Measurements were performed before and at least 10 min after administration of 0.2 mmol/kg Gadoteric acid (Dotarem, Guerbet, Paris, France) (28). T_2_ mapping was performed on the same three short-axis slices before contrast administration using a T_2_-prepared SSFP sequence with different T_2_-preparation times (0, 30, and 55 ms) (14).

### Image Analysis

All image post-processing was performed on cvi42 (Circle Cardiovascular Imaging, Calgary, Canada). Cardiac function analysis was performed by contour-tracing the short-axis cines according to previously published instructions (29).

Native and post-contrast T_1_ maps were generated for each short-axis slice using the motion-corrected images with different inversion times. T_2_ maps were generated using motion-corrected images with different echo times. All generated maps were manually segmented and reported according to the 16-segment American Heart Association model (14). Apical segments were excluded from the global analysis because of the increased risk of artifacts (30).

### Statistical Analysis

Statistical analysis was performed using SPSS (version 24.0, IBM Corp., Armonk, NY, USA). Continuous variables were presented as mean ± SD.

Multivariate linear regression models were created for LV mass, LV and right ventricular (RV) end-diastolic volume (EDV), stroke volume (SV), EF, native T_1_, ECV, and T_2_. As LV and RV SV are similar in subjects without valvular leakage, their corresponding models are also similar (10), and therefore we did not report the model of the RV SV. Each model was initially fitted using the non-modifiable-independent clinical variables age, gender, and height. Height was used to scale for body size instead of body surface area to prevent from underestimating the effect of overweight with BMI (10, 11). The initial models were then expanded by adding the independent clinical variables related to cardiovascular risk factors, including BMI, waist-hip ratio, systolic (SBP) and diastolic blood pressure (DBP), and HbA1c. Heart rate (HR) was also included in the full model as it is often increased in high-risk populations and also is an additional risk factor for cardiovascular disease (31). Weight was not included as an independent variable, because of the correlation with height. In the full models, the ß-coefficient represents the change in a dependent variable given a change of one SD in an independent variable, with SD calculated based on the entire study population. Values of *p* below 0.05 were considered statistically significant.

The percentage of variation in each cardiac MRI outcome that was explained by the full model was indicated with the coefficient of determination, i.e., the R^2^ value (32). Subsequently, the R^2^ value of the full model was reduced with the R^2^ value of the initial model to indicate the percentage of variation that was explained by cardiovascular risk factors in addition to the non-modifiable variables. For each significant independent variable in the full model, the semi-partial correlation coefficient was squared to calculate the unique variance explained by that variable (32). The presence of multicollinearity between independent variables was assessed using Pearson correlation (>0.8) and the variance inflation factor (>5) (33).

## Results

### Study Population Characteristics

**Figure 1:**
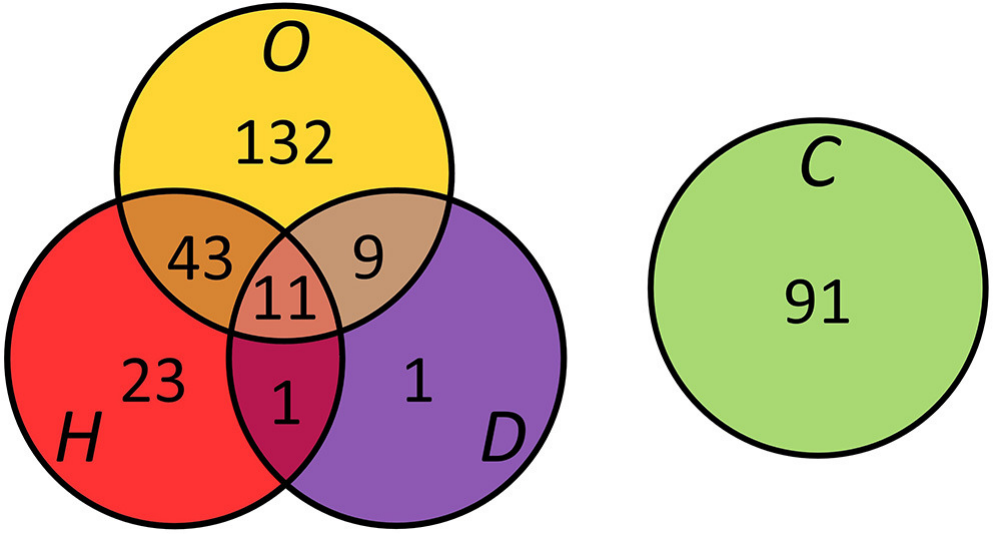
Presence and overlap of overweight (O), hypertension (H), and type 2 diabetes (D) in the study population (n = 311). Controls (C) were free of risk factors.

Three hundred and twenty-two subjects were recruited for participation. Eleven subjects were excluded from analysis as the cardiac MRI examination was unsuccessful due to either claustrophobia or scanner bore size limitations. Three hundred and eleven subjects (age, 32 ± 7 years; men, 49%) were included (Table 1). Of them, 91 subjects were free of risk factors (i.e., controls), and 220 subjects had at least one risk factor. The presence and overlap of cardiovascular risk factors are summarized in Table 1 and visualized in Figure 1. All MRI scans were screened for hemodynamically significant valvular abnormalities on all acquired long-axis cines and also for the presence of late gadolinium enhancement, none were observed. Subjects with either two or three risk factors showed higher HRs than subjects without or with one risk factor (80 ± 13 vs. 69 ± 11 bpm, *p* < 0.01). Additional cardiac MRI morphology, function, and tissue characteristics per gender are provided in Supplementary Table S2, and gender-specific per risk factor in Supplementary Tables S3, S4. The univariate analyses of independent clinical variables on cardiac MRI values are reported in Supplementary Tables S5–S14. Multicollinearity between independent variables was not demonstrated (Supplementary Tables S15, S16).

**Table 1:** Study population characteristics.

		**Males (*n* = 151)**	**Females (*n* = 160)**
Age (years)		32.6 ± 6.3	31.5 ± 7.1
Height (cm)		184 ± 7	171 ± 6
Weight (kg)		91 ± 17	82 ± 17
Body mass index (kg/m^2^)		26.9 ± 4.9	28.0 ± 5.7
Body surface area (m^2^)		2.13 ± 0.20	1.94 ± 0.19
Lean body mass (kg)		64 ± 9	47 ± 6
Waist size (cm)		98 ± 13	95 ± 15
Hip size (cm)		102 ± 9	108 ± 12
Waist–hip ratio		0.95 ± 0.07	0.89 ± 0.07
Systolic blood pressure (mmHg)		130 ± 13	125 ± 15
Diastolic blood pressure (mmHg)		84 ± 9	82 ± 10
Hemoglobin A1c (mmol/mol)		35.1 ± 9.1	34.7 ± 8.9
Glucose (mmol/L)		5.9 ± 1.8	5.7 ± 1.6
Haematocrit (%)		44.1 ± 2.4	39.5 ± 2.5
Heart rate (bpm)		69 ± 11	70 ± 11
Risk factors*	Overweight	91 (60%)	104 (65%)
	Hypertension with medication	21 (14%)	26 (16%)
	Hypertension without medication	18 (12%)	13 (8%)
	Type 2 diabetes with medication	10 (7%)	11 (7%)
	Type 2 diabetes without medication	1 (1%)	0 (0%)
	None (controls)	44 (29%)	47 (29%)

Data presented as mean ± SD or n (%). *Overlap of risk factors is visualized in Figure 1.

### LV Morphology and Function

**Figure 2:**
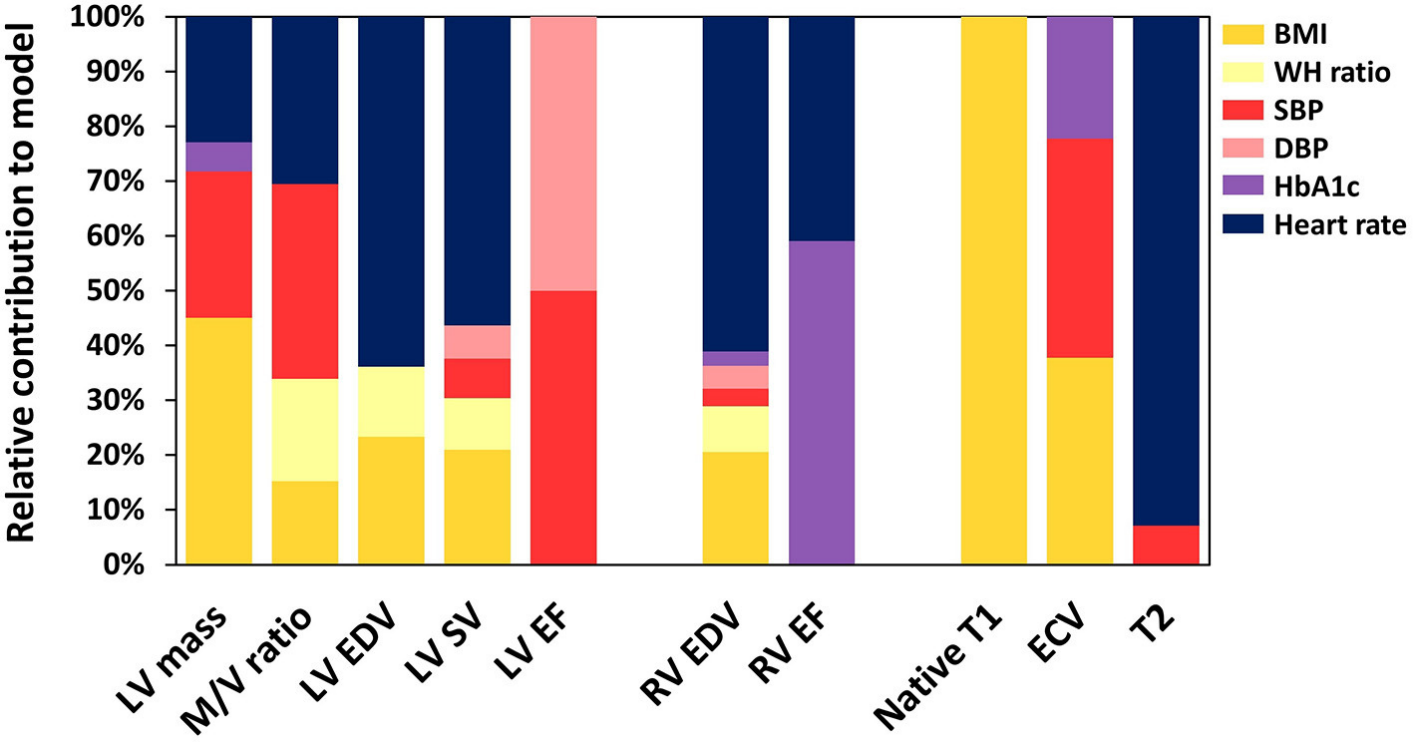
The relative contribution of risk factors to the explanation of variation in cardiac MRI results. LV, left ventricle; M/V, mass/volume; EDV, end-diastolic volume; SV, stroke volume; EF, ejection fraction; RV, right ventricle; ECV, extracellular volume; BMI, body mass index; WH, waisthip; SBP, systolic blood pressure; DBP, diastolic blood pressure.

The multivariate linear regression models for cardiac morphology and function are reported in Table 2. The contribution of independent variables to the variation in cardiac MRI outcomes is visualized in Figure 2 and Supplementary Figure S1. Higher LV mass was independently associated with increasing BMI (ß = 7.3 g per SD (5.3 kg/m^2^), *p* < 0.001), SBP (ß = 6.9 g per SD (14 mmHg), *p* < 0.001), and HbA1c (ß = 2.2 g per SD (9.0 mmol/mol), *p* < 0.05). LV mass was lower with increasing HR (ß = −4.5 g per SD (11 bpm), *p* < 0.001), while waist-hip ratio and DBP had no impact on LV mass. Among all ventricular parameters, the full model explained the most variation in LV mass of which 18% was added by risk factors.

**Table 2:** Multivariate linear regression models for cardiac morphology and function.

			**Left ventricle**					**Right ventricle**	
			**Mass**	Mass/volume ratio (^*^100)	**End-diastolic volume**	**Stroke volume**	**Ejection fraction**	**End-diastolic volume**	**Ejection fraction**
**Variable**		**Per SD**	**Change (g)**	**Change (g/ml)**	**Change (ml)**	**Change (ml)**	**Change (%)**	**Change (ml)**	**Change (%)**
Non-modifiable	Age	6.8 years	−2.3 (−4.1, −0.6)^†^	−0.1 (−1.3, 1.1)	−3.0 (−5.8, −0.1)^*^	−0.6 (−2.5, 1.4)	0.7 (0.2, 1.3)^*^	−4.6 (−7.8, −1.3)^†^	0.8 (0.3, 1.4)^†^
	Gender	Female	−20 (−25, −16)^‡^	−9.7 (−12.9, −6.6)^‡^	−10.4 (−17.9, −3.0)^†^	−3.1 (−8.2, 1.9)	1.9 (0.3, 3.4)^*^	−19 (−28, −11)^‡^	3.8 (2.4, 5.2)^‡^
	Height	9.0 cm	8.4 (6.3, 10.5)^‡^	−2.2 (−3.7, −0.8)^†^	19 (16, 23)^‡^	11.3 (8.9, 13.6) ^‡^	−0.1 (−0.7, 0.6)	22 (18, 25)^‡^	0.2 (−0.4, 0.9)
Risk factors	BMI	5.3 kg/m^2^	7.3 (5.4, 9.0)^‡^	1.5 (0.2, 2.8)^*^	8.6 (5.4, 11.7)^‡^	5.0 (2.9, 7.1)^‡^	−0.0 (−0.6, 0.6)	9.9 (6.4, 13.4)^‡^	−0.2 (−0.7, 0.4)
	WHR	0.078	−0.8 (−2.9, 1.4)	1.9 (0.4, 3.4)^*^	−7.5 (−11.1, −3.8)^‡^	−4.0 (−6.5, −1.5)^†^	0.2 (−0.5, 0.9)	−7.5 (−11.6, −3.4)^‡^	−0.2 (−0.8, 0.5)
	SBP	14 mmHg	6.9 (4.6, 9.2)^‡^	2.8 (1.2, 4.5)^‡^	3.6 (−0.2, 7.5)	3.7 (1.1, 6.4)^†^	1.0 (0.2, 1.8)^*^	4.9 (0.6, 9.3)^*^	0.5 (−0.2, 1.3)
	DBP	10 mmHg	0.8 (−1.6, 3.2)	1.7 (−0.1, 3.4)	−3.0 (−7.0, 1.0)	−3.6 (−6.3, −0.8)^*^	−1.1 (−1.9, −0.2)^*^	−5.7 (−10.2, −1.1)^*^	−0.2 (−1.0, 0.5)
	HbA1c	9.0 mmol/mol	2.2 (0.5, 3.8)^*^	1.0 (−0.1, 2.2)	1.3 (−1.4, 4.0)	0.0 (−1.8, 1.9)	−0.5 (−1.0, 0.1)	3.2 (0.1, 6.3)^*^	−0.8 (−1.3, −0.3)^†^
	HR	11 bpm	−4.5 (−6.1, −2.9)^‡^	1.8 (0.7, 2.9)^†^	−12.2 (−14.9, −9.6)^‡^	−7.1 (−8.9, −5.3)^‡^	0.1 (−0.5, 0.6)	−15 (−18, −12)^‡^	0.7 (0.1, 1.2)^*^
Variation explained by (R^2^)	Non-modifiable variables		50.5%	19.9%	40.3%	31.7%	3.2%	45.2%	15.6%
	Risk factors		18.4%	23.5%	16.1%	14.9%	1.4%	16.1%	3.8%
	Full model		68.9%	43.4%	56.4%	46.6%	4.6%	61.3%	19.4%
	Unknown variables		31.1%	56.6%	43.6%	53.4%	95.4%	38.7%	80.6%

*Change in cardiac MRI value, noted as mean (95% CI), given a change of one standard deviation in the independent variable. Level of significance:*

* *p < 0.05,*

† *p < 0.01,*

‡ *p < 0.001. SD, standard deviation; BMI, body mass index; WHR, waist-hip ratio; SBP, systolic blood pressure; DBP, diastolic blood pressure; HR, heart rate*.

Higher LV mass/volume ratio was associated with increasing BMI (ß = 0.015 g/ml per SD, *p* < 0.05), waist-hip ratio (ß = 0.019 g/ml per SD (0.078), *p* < 0.05), SBP (ß = 0.028 g/ml per SD, *p* < 0.001), and HR (ß = 0.018 g/ml per SD, *p* < 0.01). Associations with DBP (*p* = 0.06) and HbA1c (*p* = 0.08) were not significant. Compared to other ventricular parameters, cardiovascular risk factors explained the most variation in the LV mass/volume ratio (R^2^ = 24%).

Higher LV EDV was associated with increasing BMI (ß = 8.6 ml per SD, *p* < 0.001). LV EDV was lower with increasing waist-hip ratio (ß = −7.5 ml per SD, *p* < 0.001), and HR (ß = −12.2 ml per SD, *p* < 0.001). Furthermore, the association between LV EDV and SBP was not significant (*p* = 0.06), while DBP and HbA1c also had no impact.

Higher LV SV was associated with increasing BMI (ß = 5.0 ml per SD, *p* < 0.001) and SBP (ß = 3.7 ml per SD, *p* < 0.01). LV SV was lower with increasing waist-hip ratio (ß = −4.0 ml per SD, *p* < 0.01), DBP (ß = −3.6 ml per SD (10 mmHg), *p* < 0.05), and HR (ß = −7.1 ml per SD, *p* < 0.001). HbA1c had no impact on LV SV. The LV EF was only associated with SBP (ß = 1.0% per SD, *p* < 0.05) and DBP (ß = −1.1% per SD, *p* < 0.05). The variation explained in LV EF by the full model (R^2^ = 5%) was the lowest among all ventricular parameters.

### RV Morphology and Function

Similar to the LV, the RV EDV was higher with increasing BMI (ß = 9.9 ml per SD, *p* < 0.001) and lower with increasing waist-hip ratio (ß = −7.4 ml per SD, *p* < 0.001) and HR (ß = −15 ml per SD, *p* < 0.001; Table 2). Conversely to the LV, the RV EDV was also higher with increasing SBP (ß = 4.9 ml per SD, *p* < 0.05) and HbA1c (ß = 3.2 ml per SD, *p* < 0.05), while RV EDV was lower with increasing DBP (ß = −5.7 ml per SD, *p* < 0.05).

The RV EF was positively associated with HR (ß = 0.7% per SD, *p* < 0.05) and negatively with HbA1c (ß = −0.8% per SD, *p* < 0.01). The other variables had no impact on the RV EF.

### Myocardial Tissue Characteristics

The multivariate linear regression models for myocardial tissue characteristics are reported in Table 3. Higher native T_1_ values were associated with increasing BMI (ß = 7.3 ms per SD, *p* < 0.01). Associations with SBP and DBP were not significant (*p* = 0.07), and all other variables had no impact. Only a little variation in native T_1_ values was explained by the full model (R^2^ = 5%).

**Table 3:** Multivariate linear regression models for myocardial tissue characteristics.

			**Native T_1_**	**Extracellular volume**	**T_2_**
**Variable**		**Per SD**	**Change (ms)**	**Change (%)**	**Change (ms)**
Non-modifiable	Age	6.8 years	−2.5 (−6.7, 1.6)	0.27 (0.03, 0.51)^†^	0.08 (−0.11, 0.27)
	Gender	Female	9.0 (−1.7, 19.6)	2.8 (2.2, 3.4)^‡^	1.15 (0.65, 1.65)^‡^
	Height	9.0 cm	7.6 (2.7, 12.6)^†^	0.18 (−0.11, 0.46)	−0.00 (−0.23, 0.23)
Risk factors	BMI	5.3 kg/m^2^	7.3 (2.9, 11.7)^†^	−0.38 (−0.64, −0.12)^†^	−0.16 (−0.37, 0.04)
	WHR	0.078	−3.9 (−9.2, 1.3)	−0.04 (−0.34, 0.26)	0.06 (−0.19, 0.30)
	SBP	14 mmHg	−5.2 (−10.7, 0.4)	−0.48 (−0.79, −0.16)^†^	−0.39 (−0.65, −0.13)^†^
	DBP	10 mmHg	5.4 (−0.4, 11.2)	−0.01 (−0.35, 0.32)	0.03 (−0.24, 0.30)
	HbA1c	9.0 mmol/mol	1.0 (−2.9, 5.0)	0.27 (0.04, 0.49)^†^	−0.12 (−0.30, 0.06)
	HR	11 bpm	3.2 (−0.6, 7.1)	−0.09 (−0.31, 0.12)	−0.95 (−1.13, −0.78)^‡^
Variation explained by (R^2^)	Non-modifiable variables		1.3%	30.9%	8.0%
	Risk factors		4.1%	7.0%	33.3%
	Full model		5.4%	37.9%	41.3%
	Unknown variables		94.6%	62.1%	58.7%

*Change in cardiac MRI value, noted as mean (95% CI), given a change of one standard deviation in the independent variable. Level of significance:*

† *p < 0.01,*

‡ *p < 0.001. SD, standard deviation; BMI, body mass index; WHR, waist-hip ratio; SBP, systolic blood pressure; DBP, diastolic blood pressure; HR, heart rate*.

Higher ECV was associated with increasing HbA1c (ß = 0.27% per SD, *p* < 0.01). ECV was lower with increasing BMI (ß = −0.38% per SD, *p* < 0.01) and SBP (ß = −0.48% per SD, *p* < 0.01). The full model explained 38% of the variation in ECV, of which 7% was added by cardiovascular risk factors.

T_2_ values were lower with increasing SBP (ß = −0.39 ms per SD, *p* < 0.01) and HR (ß = −0.95 ms per SD, *p* < 0.001). All other variables had no impact on T_2_ values. Cardiovascular risk factors explained 33% of the variation in T_2_ values (full model R^2^ = 41%).

## Discussion

In this cross-sectional cardiac MRI study, we demonstrated that cardiovascular risk factors in an asymptomatic young adult cohort are associated with subclinical cardiac alterations. Substantial variation in LV mass, LV mass/volume ratio, cardiac volumes, ECV, and T_2_ values was explained by independent clinical variables related to overweight, hypertension, and T2D. Variation in EF and native T_1_ values, however, was explained to a lesser extent.

With multivariate regression analysis, we showed that increased BMI, SBP, and HbA1c are independently associated with higher LV mass in young adults. The independent linkage of BMI and SBP on LV mass caused by respectively volume and pressure overload was in agreement with previous cardiac MRI findings in mature adult populations (age range, 41–85 years) (10–12) and an echocardiographic study in adolescents (mean age, 16 ± 2 years) that reported increasing LV mass with increasing blood pressure (34). The positive association between HbA1c and LV mass was in line with results in mature study cohorts (11, 35), but also with echocardiographic findings in young adults (mean age, 27 ± 7 years) (36). In the current study, risk factors explained less variation in LV mass compared to studies in mature populations (age range, 45–85 years) (10, 11). This difference might be age-related, as young adults are more likely to be exposed to these risk factors for a shorter period. Furthermore, in asymptomatic mature populations, increased LV mass is linked with heart failure events (37). This deleterious effect of increased LV mass could also occur in the growing young adult risk population, possibly worsening when conditions remain unchanged later in life.

Cardiac and SVs were significantly associated with BMI, waist-hip ratio, blood pressure, and HR, while associations with HbA1c were less obvious. In previous studies, being overweight was linked to chamber dilatation with BMI as an independent predictor (10–12, 38). Although BMI is the gold standard to define overweight, it does not reflect on the actual body fat distribution which strongly correlates with cardiovascular risk, therefore we also included waist-hip ratio as an independent variable (39). As expected, increased BMI was associated with higher cardiac volumes, but surprisingly, a higher waist*-*hip ratio was associated with lower cardiac volumes and increased mass/volume ratio. These results confirm that especially central body fat deposition is associated with adverse concentric remodeling (40). We also found that cardiac volumes are lower with increased HR, confirming results in a previous study (38). Another study hypothesized that increased resting HR contributes to initial cardiac remodeling, requiring smaller cardiac volumes, and this possibly also applies to our young population (41). The opposite effect of SBP and DBP on end-diastolic and SVs was also in concordance with previous findings in mature populations (10–12). In this study, HbA1c only showed a positive association with RV volumes, while no impact on the LV was found which confirmed findings in a previous study in young T2D subjects (aged 32 ± 7 years) that only examined the LV (4). In mature populations, however, T2D was associated with smaller LV and RV volumes (10, 11), suggesting that the impact of T2D on cardiac volumes could be age-related. As the aforementioned alterations in cardiac dimensions can overlap early findings in dilated cardiomyopathy, these need to be considered in imaging diagnostics (9).

The full models for LV and RV EF only explained respectively 5 and 20% of the existing variation. These low percentages, combined with the substantially higher explained variation for RV relative to LV function, confirmed regression models previously reported in mature populations (10–12). Similar to these studies, we showed that overweight-related variables had no impact on the EF (10–12). We also showed that SBP had a positive association, while DBP and HbA1c were negatively associated with EF. These results are in line with findings in older adult populations, suggesting that hypertension and T2D cause subclinical systolic changes irrespective of age.

To our best knowledge, this is the first study that explained variation in myocardial tissue characteristics (including T_1_, T_2_, and ECV) using multivariate regression analysis with cardiovascular risk factor-related predictors. BMI showed a significant association with native T_1_ values, while previous studies showed similar T_1_ values in both overweight (aged 41 ± 13 years) and obese subjects (aged 32 ± 7 years) relative to normal weights (22, 42). Comparable to our results, T2D populations had similar T_1_ values relative to controls (21). Previous studies reported increased T_1_ values in hypertensive subjects with LVH (15). We found no such association, which is probably related to the relatively small LV mass increase in our hypertensives.

Current results indicate that increased BMI and blood pressure are associated with reduced ECV, suggesting hypertrophy of myocytes similar as seen in athletes (43). Conversely, ECV is often increased in non-ischemic cardiomyopathies (14). ECV could therefore potentially discriminate adaptations associated with overweight, obesity, or hypertension from early pathology, such as previously suggested to distinguish the athlete's heart from HCM (20, 44). The positive association between HbA1c and ECV was in agreement with findings in subjects with T2D (21). Increased ECV is linked to heart failure (45), underlining the importance of its early recognition.

Heart rate was the most significant negatively associated predictor of T_2_ values. This can be explained by the nature of the T_2_-prepared SSFP sequence, which can produce incomplete T_1_ relaxation during increased HRs, influencing T_2_ values (46). Individuals with cardiovascular risk factors often have increased HRs, as confirmed in the current study (31). Using standard T_2_-prepared SSFP sequences could therefore result in underestimation of true T_2_ values in these populations, which needs to be taken into consideration (28).

Increased SBP was the only other independently associated predictor of (lower) T_2_ values. Our findings confirm similar T_2_ values reported for normal-weight and overweight subgroups (age 41 ± 13 years) (42), however, they are in contrast with studies reporting higher T_2_ values in T2D populations (aged 56 ± 12 years) and hypertensives with LVH (aged 54 ± 16 years) (24, 25). Most likely, in the latter group, higher T_2_ values are caused by the used scanning techniques, FLASH, which is not susceptible to HR effects. Discrepancies might be explained by longer exposure to risk factors causing T_2_ alterations similar to those found in non-ischemic cardiomyopathies (16).

### Limitations

Firstly, we did not measure the metabolic risk factors triglyceride and cholesterol (47), therefore their impact on cardiac outcomes was not tested. Nevertheless, as previously reported in mature populations, these risk factors have substantially less impact on the heart than the risk factors included in our study (10–12). Secondly, in this young asymptomatic population, we could not link the demonstrated subclinical cardiac alterations with long-term adverse events due to the cross-sectional study design. Longitudinal studies on young high-risk populations may provide insights on this matter. Thirdly, our prospectively included young adult population was relatively small compared to mature populations in previous studies using a retrospective study design (10–12). Nevertheless, our population size allowed for appropriate multivariate linear regression (48).

## Conclusion

In summary, we showed in an asymptomatic young adult population that sedentary lifestyle-related risk factors are associated with subclinical cardiac alterations on MRI, and this needs to be considered in clinical decision-making. These findings are also comparable to studies in more mature populations, which already demonstrated that subclinical cardiac alterations are associated with long-term adverse cardiac events. If current trends in risk factor prevalence keep rising, this may cause additional cardiovascular disease burden, especially in Westernized countries.

## Data Availability Statement

The original contributions presented in the study are included in the article Supplementary Material, further inquiries can be directed to the corresponding author.

## Ethics Statement

The studies involving human participants were reviewed and approved by Medical Ethics Committee of the University Medical Center Groningen (no. 2016/476). The patients/participants provided their written informed consent to participate in this study.

## Author Contributions

VD, RD, BV, and NP conceptualized the study. GS, MB, KH-O, and NP drafted the manuscript. GS, MB, RB, and NP contributed in data acquisition. GS and NP interpreted the data. RS, RD, and NP supervised the project. RD, RB, and NP were responsible for resources. RS, VD, CN, DS, and BV were responsible for review and editing. All authors approved the submitted manuscript.

## Funding

This work was supported by the Dutch Heart Association (2016T042).

## Conflict of Interest

The authors declare that the research was conducted in the absence of any commercial or financial relationships that could be construed as a potential conflict of interest.

## Publisher's Note

All claims expressed in this article are solely those of the authors and do not necessarily represent those of their affiliated organizations, or those of the publisher, the editors and the reviewers. Any product that may be evaluated in this article, or claim that may be made by its manufacturer, is not guaranteed or endorsed by the publisher.

## Abbreviations

BMI: body mass index
DBP: diastolic blood pressure
ECV: extracellular volume
EDV: end-diastolic volume
EF: ejection fraction
HbA1c: hemoglobin A1c
HCM: hypertrophic cardiomyopathy
LV: left ventricle
LVH: left ventricular hypertrophy
MRI: magnetic resonance imaging
RV: right ventricle
SBP: systolic blood pressure
SD: standard deviation
SSFP: steady-state free precession
SV: stroke volume
T2D: type 2 diabetes
HR: heart rate.
