# Supplementary material for: Cardiac Alterations on 3T MRI in Young Adults With Sedentary Lifestyle-Related Risk Factors
Source: Front Cardiovasc Med. 2022 Feb 22;9:840790. doi: 10.3389/fcvm.2022.840790 (PMC8902075; doi:10.3389/fcvm.2022.840790)
Supplement: Supplementary file 1 [file Data_Sheet_1.pdf]

Supplementary Material

1 Supplementary Figures and Tables

1.1 Supplementary Figure

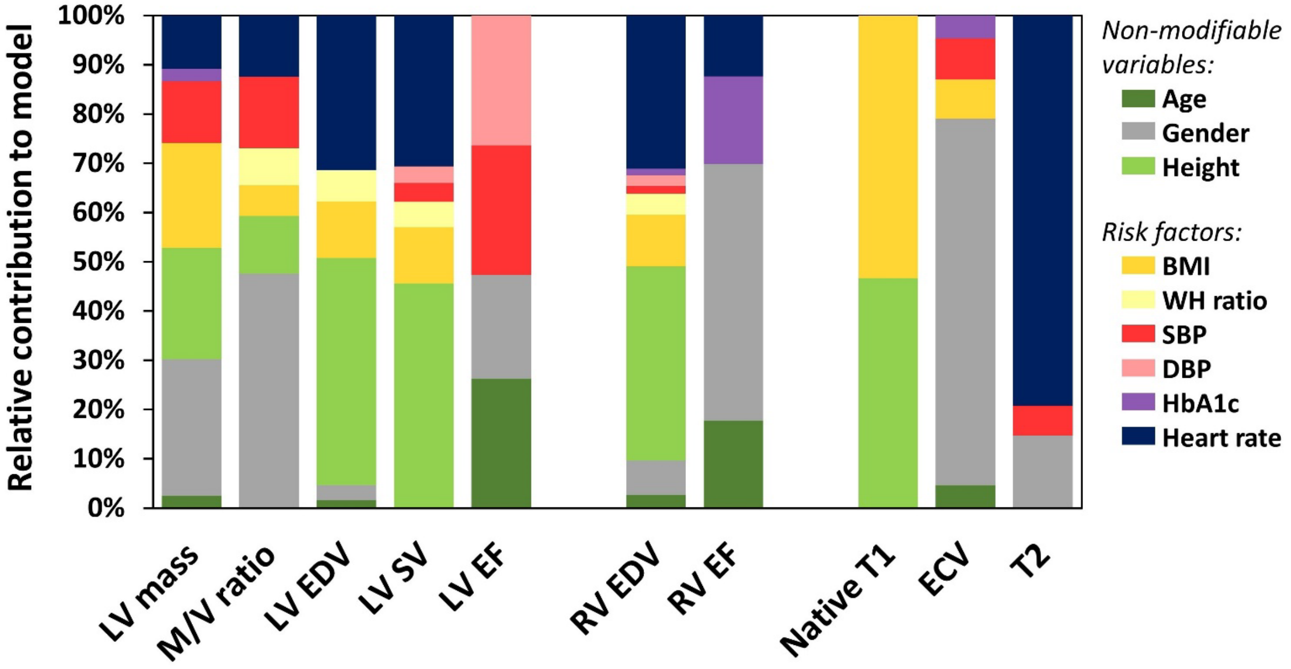

Supplementary Figure 1. Relative contribution of non-modifiable variables and risk factors to the explanation of variation in cardiac MRI results. *LV* left ventricle, *M/V* mass/volume, *EDV* end-diastolic volume, *SV* stroke volume, *EF* ejection fraction, *RV* right ventricle, *ECV* extracellular volume, *BMI* body mass index, *WH* waist–hip, *SBP* systolic blood pressure, *DBP* diastolic blood pressure.

1.2 Supplementary Tables

Supplementary Table 1. CMR acquisition parameters.

	**Short-axis cine**	**Native T_1**	**Post-contrast T_1**	**T_2**
**Repetition time (ms)**	38.92–44.52	248.16–303.59	314.60–352.16	169.59–235.74
**Echo time (ms)**	1.15–1.31	1.05–1.12	1.01	1.28–1.32
**Flip angle (º)**	43−46	35	35	12
**Field of view (mm^2)**	300–453 x 225–453	360–428 x 202–428	360–428 x 202–428	202–428 x 360–428
**Matrix**	256 x 192–256	256 x 144–218	192 x 108–192	192 x 108–192
**Pixel bandwidth (Hz/pixel)**	930	1085	1085	1185

Supplementary Table 2. Cardiac MRI values per gender.

		**Males** *n* = 151	**Females** *n* = 160	***P***
**Left ventricle**	**Mass** (g)	117 ± 20	85 ± 16	<0.001
	**LV mass/volume ratio** (g/ml)	0.65 ± 0.13	0.56 ± 0.11	<0.001
	**End-diastolic volume** (ml)	185 ± 35	155 ± 24	<0.001
	**Stroke volume** (ml)	109 ± 22	93 ± 16	<0.001
	**Ejection fraction** (%)	59 ± 5	61 ± 4	<0.01
	**Indexed mass** (g/m^2)	55 ± 8	44 ± 7	<0.001
	**Indexed end-diastolic volume** (ml/m^2)	87 ± 16	80 ± 12	<0.001
	**Indexed stroke volume** (ml/m^2)	52 ± 10	48 ± 8	<0.001
**Right ventricle**	**End-diastolic volume** (ml)	213 ± 40	170 ± 28	<0.001
	**Ejection fraction** (%)	51 ± 4	55 ± 4	<0.001
	**Indexed end-diastolic volume** (ml/m^2)	100 ± 18	88 ± 14	<0.001
**Tissue characteristics**	**Native** T_1 (ms)	1148 ± 29	1153 ± 37	0.21
	**Extracellular volume** (%)	22 ± 2	25 ± 2	<0.001
	T_2 (ms)	38 ± 2	39 ± 2	<0.001

Indexed for body surface area

Supplementary Table 3. Cardiac MRI values on risk factors in males.

		Controls<br>*n* = 44	Overweight<br>*n* = 63	Hypertensive<br>*n* = 15	Overweight & hypertensive<br>*n* = 18
LV	**Mass** (g)	107 ± 16 (133)	118 ± 16 (144)	127 ± 24 (166)	127 ± 24 (166)
	**M/V ratio** (g/ml)	0.58 ± 0.10 (0.74)	0.66 ± 0.11 (0.84)	0.63 ± 0.07 (0.75)	0.71 ± 0.10 (0.88)
	**EDV** (ml)	188 ± 33 (242)	183 ± 34 (239)	203 ± 32 (256)	181 ± 39 (245)
	**ESV** (ml)	77 ± 16 (103)	75 ± 16 (101)	85 ± 16 (111)	72 ± 19 (103)
	**SV** (ml)	111 ± 22 (147)	107 ± 21 (142)	117 ± 18 (147)	109 ± 23 (147)
	**CO** (l/min)	7.3 ± 1.6 (9.9)	7.3 ± 1.5 (9.8)	7.6 ± 1.0 (9.2)	8.2 ± 1.6 (10.8)
	**EF** (%)	59 ± 5 (67)	59 ± 5 (67)	58 ± 4 (65)	60 ± 5 (68)
	**Mass/H$^{2.7}$** (g/m$^{2.7}$)	20.7 ± 3.2 (26.0)	23.2 ± 2.9 (28.0)	23.1 ± 3.9 (29.5)	24.3 ± 4.3 (31.4)
	**EDV/H$^{2.7}$** (ml/m$^{2.7}$)	36.2 ± 5.3 (44.9)	36.0 ± 6.1 (46.0)	36.9 ± 5.0 (45.1)	34.5 ± 6.5 (45.2)
	**ESV/H$^{2.7}$** (ml/m$^{2.7}$)	14.8 ± 2.8 (19.4)	14.8 ± 3.0 (19.7)	15.5 ± 2.6 (19.8)	13.8 ± 3.2 (19.1)
	**SV/H$^{2.7}$** (ml/m$^{2.7}$)	21.4 ± 3.5 (27.2)	21.1 ± 3.9 (27.5)	21.4 ± 3.1 (26.5)	20.8 ± 4.1 (27.5)
	**CO/H$^{2.7}$** (l/min/m$^{2.7}$)	1.39 ± 0.26 (1.82)	1.43 ± 0.29 (1.91)	1.39 ± 0.17 (1.67)	1.56 ± 0.27 (2.00)
RV	**EDV** (ml)	215 ± 35 (273)	211 ± 41 (278)	231 ± 40 (297)	206 ± 40 (272)
	**ESV** (ml)	104 ± 18 (134)	104 ± 23 (142)	113 ± 23 (151)	97 ± 21 (132)
	**SV** (ml)	111 ± 21 (146)	107 ± 21 (142)	119 ± 22 (155)	109 ± 22 (145)
	**CO** (l/min)	7.2 ± 1.5 (9.7)	7.3 ± 1.5 (9.8)	7.7 ± 1.3 (9.8)	8.2 ± 1.6 (10.8)
	**EF** (%)	51 ± 4 (58)	51 ± 4 (58)	51 ± 4 (58)	53 ± 4 (60)
	**EDV/H$^{2.7}$** (ml/m$^{2.7}$)	41.4 ± 5.6 (50.6)	41.5 ± 7.4 (53.7)	42.0 ± 5.7 (51.4)	39.4 ± 7.0 (50.9)
	**ESV/H$^{2.7}$** (ml/m$^{2.7}$)	20.1 ± 3.2 (25.4)	20.4 ± 4.3 (27.5)	20.4 ± 3.4 (26.0)	18.5 ± 3.6 (24.4)
	**SV/H$^{2.7}$** (ml/m$^{2.7}$)	21.3 ± 3.3 (26.7)	21.1 ± 3.9 (27.5)	21.5 ± 3.4 (27.1)	20.9 ± 4.1 (27.6)
	**CO/H$^{2.7}$** (l/min/m$^{2.7}$)	1.39 ± 0.26 (1.82)	1.43 ± 0.28 (1.89)	1.40 ± 0.18 (1.70)	1.57 ± 0.28 (2.03)
**Native T$_1$** (ms)		1140 ± 25 (1181)	1153 ± 25 (1194)	1147 ± 28 (1193)	1146 ± 28 (1192)
**Extracellular volume** (%)		22.8 ± 1.5 (25.3)	21.9 ± 1.9 (25.0)	22.8 ± 1.4 (25.1)	21.7 ± 1.3 (23.8)
**T$_2$** (ms)		38.2 ± 1.7 (41.0)	37.5 ± 1.5 (40.0)	37.9 ± 1.3 (40.0)	36.8 ± 2.0 (40.1)

Data noted as mean ± standard deviation (95th percentile). Indexation by height to the power of 2.7 (H$^{2.7}$) instead of body surface area to avoid masking the effect of overweight. Cardiac MRI values in type 2 diabetes populations were not reported due to small group size. *LV* left ventricle, *M/V* mass/volume, *EDV* end-diastolic volume, *ESV* end-systolic volume, *SV* stroke volume, *EF* ejection fraction, *RV* right ventricle.

Supplementary Table 4. Cardiac MRI values on risk factors in females.

		Controls<br>*n* = 47	Overweight<br>*n* = 69	Hypertensive<br>*n* = 8	Overweight & hypertensive<br>*n* = 25
LV	**Mass** (g)	75 ± 11 (93)	86 ± 13 (107)	94 ± 15 (119)	95 ± 18 (125)
	**M/V ratio** (g/ml)	0.49 ± 0.06 (0.59)	0.54 ± 0.07 (0.66)	0.69 ± 0.18 (0.99)	0.64 ± 0.09 (0.79)
	**EDV** (ml)	156 ± 20 (189)	159 ± 24 (198)	139 ± 23 (177)	150 ± 28 (196)
	**ESV** (ml)	63 ± 11 (81)	62 ± 11 (80)	55 ± 8 (68)	58 ± 16 (84)
	**SV** (ml)	93 ± 14 (116)	97 ± 16 (123)	84 ± 17 (112)	92 ± 15 (117)
	**CO** (l)	6.2 ± 1.1 (8.0)	6.7 ± 1.3 (8.8)	5.7 ± 1.1 (7.5)	6.8 ± .9 (8.3)
	**EF** (%)	60 ± 5 (68)	61 ± 4 (68)	60 ± 4 (67)	62 ± 5 (70)
	**Mass/$H^{2.7}$** ($g/m^{2.7}$)	17.3 ± 2.8 (21.9)	20.2 ± 3.1 (25.3)	21.5 ± 2.9 (26.3)	22.4 ± 4.2 (29.3)
	**EDV/$H^{2.7}$** ($ml/m^{2.7}$)	35.7 ± 4.6 (43.3)	37.4 ± 5.1 (45.8)	32.0 ± 5.0 (40.3)	35.5 ± 6.6 (46.4)
	**ESV/$H^{2.7}$** ($ml/m^{2.7}$)	14.4 ± 2.5 (18.5)	14.7 ± 2.7 (19.1)	12.6 ± 1.7 (15.4)	13.7 ± 3.8 (20.0)
	**SV/$H^{2.7}$** ($ml/m^{2.7}$)	21.3 ± 3.2 (26.6)	22.7 ± 3.3 (28.1)	19.4 ± 3.8 (25.7)	21.7 ± 3.3 (27.1)
	**CO/$H^{2.7}$** ($l/m^{2.7}$)	1.41 ± 0.24 (1.80)	1.58 ± 0.28 (2.04)	1.31 ± 0.24 (1.70)	1.61 ± 0.22 (1.97)
RV	**EDV** (ml)	173 ± 22 (209)	176 ± 28 (222)	149 ± 23 (187)	159 ± 29 (207)
	**ESV** (ml)	79 ± 13 (100)	80 ± 16 (106)	64 ± 11 (82)	69 ± 16 (95)
	**SV** (ml)	94 ± 14 (117)	96 ± 17 (124)	85 ± 17 (113)	91 ± 15 (116)
	**CO** (l)	6.2 ± 1.1 (8.0)	6.7 ± 1.3 (8.8)	5.7 ± 1.1 (7.5)	6.7 ± .9 (8.2)
	**EF** (%)	54 ± 4 (61)	55 ± 4 (62)	57 ± 5 (65)	57 ± 4 (64)
	**EDV/$H^{2.7}$** ($ml/m^{2.7}$)	39.6 ± 5.3 (48.3)	41.4 ± 6.2 (51.6)	34.3 ± 4.7 (42.0)	37.7 ± 6.6 (48.6)
	**ESV/$H^{2.7}$** ($ml/m^{2.7}$)	18.2 ± 3.0 (23.1)	18.8 ± 3.8 (25.0)	14.8 ± 2.3 (18.6)	16.2 ± 3.8 (22.4)
	**SV/$H^{2.7}$** ($ml/m^{2.7}$)	21.5 ± 3.3 (26.9)	22.6 ± 3.4 (28.2)	19.5 ± 3.7 (25.6)	21.4 ± 3.3 (26.8)
	**CO/$H^{2.7}$** ($l/m^{2.7}$)	1.42 ± 0.24 (1.81)	1.58 ± 0.28 (2.04)	1.31 ± 0.23 (1.69)	1.59 ± 0.22 (1.95)
**Native T_1** (ms)		1150 ± 37 (1211)	1153 ± 35 (1211)	1153 ± 41 (1220)	1156 ± 44 (1228)
**Extracellular volume** (%)		25.8 ± 2.1 (29.3)	24.9 ± 2.0 (28.2)	24.5 ± 1.9 (27.6)	24.5 ± 2.2 (28.1)
**T_2** (ms)		39.6 ± 1.8 (42.6)	38.8 ± 2.0 (42.1)	38.5 ± 1.6 (41.1)	38.0 ± 2.3 (41.8)

Data noted as mean ± standard deviation (95th percentile). Indexation by height to the power of 2.7 ($H^{2.7}$) instead of body surface area to avoid masking the effect of overweight. Cardiac MRI values in type 2 diabetes populations were not reported due to small group size. *LV* left ventricle, *M/V* mass/volume, *EDV* end-diastolic volume, *ESV* end-systolic volume, *SV* stroke volume, *CO* cardiac output, *EF* ejection fraction, *RV* right ventricle.

Supplementary Table 5. Univariate demographic predictors of LV mass.

		Males		Females		Both genders	
**Variable**	**Per**	*ß*	*r*	*ß*	*r*	*ß*	*r*
**Age**	6.8 years	1.03	0.048	1.89	0.128	2.9	0.118*
**Gender**	Female	N/A	N/A	N/A	N/A	−32	0.671‡
**Height**	9.0 cm	10.0	0.405‡	4.7	0.196*	15.2	0.634‡
**Weight**	18 kg	9.6	0.468‡	8.0	0.496‡	12.2	0.505‡
**Body mass index**	5.3 kg/m^2	6.6	0.302‡	6.5	0.442‡	4.8	0.198‡
**Body surface area**	0.21 m^2	11.4	0.531‡	9.0	0.503‡	15.4	0.641‡
**Lean body mass**	11 kg	12.9	0.507‡	14.4	0.509‡	18.1	0.749‡
**Waist size**	14 cm	7.8	0.362‡	5.7	0.389‡	8.1	0.336‡
**Hip size**	11 cm	8.3	0.339‡	5.8	0.413‡	2.2	0.093
**Waist–hip ratio**	0.078	6.5	0.306‡	2.2	0.123	10.4	0.437‡
**Systolic BP**	14 mmHg	9.5	0.430‡	6.3	0.427‡	10.1	0.418‡
**Diastolic BP**	10 mmHg	6.4	0.296‡	5.4	0.361‡	7.5	0.307‡
**HbA1c**	9.0 mmol/mol	4.4	0.212†	1.74	0.110	3.2	0.128*
**Glucose**	1.8 mmol/L	4.0	0.197*	0.70	0.044	3.1	0.127*
**Haematocrit**	3.4%	−1.41	0.049	1.05	0.051	11.2	0.462‡
**Heart rate**	11 bpm	−1.39	0.070	−1.47	0.093	−2.4	0.101
**Overweight**	Yes	8.7	0.213†	10.8	0.332‡	7.9	0.159†
**Hypertension**	Yes	15.3	0.334‡	13.5	0.373‡	14.9	0.268‡
**Diabetes**	Yes	13.0	0.163*	8.1	0.131	10.2	0.107

ß is the change in dependent variable given a change of one standard deviation in the independent variable. R is the Pearson correlation. * P<0.05; † P<0.01; ‡ P<0.001. *BP* blood pressure.

Supplementary Table 6. Univariate demographic predictors of LV mass/volume ratio.

		**Males**		**Females**		**Both genders**	
**Variable**	**Per**	*ß*	*r*	*ß*	*r*	*ß*	*r*
**Age**	6.8 years	0.031	0.224†	0.025	0.251†	0.031	0.248‡
**Gender**	Female	N/A	N/A	N/A	N/A	−0.091	0.365‡
**Height**	9.0 cm	−0.028	0.180*	−0.027	0.166*	0.016	0.131*
**Weight**	18 kg	0.039	0.300‡	0.029	0.261‡	0.042	0.342‡
**Body mass index**	5.3 kg/m^2	0.054	0.396‡	0.032	0.321‡	0.036	0.289‡
**Body surface area**	0.21 m^2	0.027	0.201*	0.024	0.196†	0.041	0.327‡
**Lean body mass**	11 kg	0.035	0.219†	0.039	0.203†	0.050	0.403‡
**Waist size**	14 cm	0.052	0.384‡	0.030	0.297‡	0.043	0.346‡
**Hip size**	11 cm	0.029	0.190*	0.023	0.198†	0.009	0.078
**Waist–hip ratio**	0.078	0.067	0.497‡	0.031	0.256‡	0.060	0.483‡
**Systolic BP**	14 mmHg	0.040	0.293‡	0.060	0.599‡	0.058	0.467‡
**Diastolic BP**	10 mmHg	0.036	0.264†	0.060	0.585‡	0.054	0.427‡
**HbA1c**	9.0 mmol/mol	0.047	0.358‡	0.026	0.244†	0.036	0.285‡
**Glucose**	1.8 mmol/L	0.034	0.268‡	0.018	0.171*	0.028	0.224‡
**Haematocrit**	3.4%	0.017	0.095	0.013	0.089	0.039	0.313‡
**Heart rate**	11 bpm	0.052	0.414‡	0.030	0.278‡	0.037	0.302‡
**Overweight**	Yes	0.098	0.380‡	0.061	0.275‡	0.074	0.287‡
**Hypertension**	Yes	0.058	0.201*	0.130	0.530‡	0.096	0.334‡
**Diabetes**	Yes	0.173	0.342‡	0.115	0.276‡	0.142	0.287‡

ß is the change in dependent variable given a change of one standard deviation in the independent variable. R is the Pearson correlation. * P<0.05; † P<0.01; ‡ P<0.001. *BP* blood pressure.

Supplementary Table 7. Univariate demographic predictors of LV end-diastolic volume.

		Males		Females		Both genders	
**Variable**	**Per**	*ß*	*r*	*ß*	*r*	*ß*	*r*
**Age**	6.8 years	−5.6	0.147	−3.3	0.143	−2.9	0.087
**Gender**	Female	N/A	N/A	N/A	N/A	−31	0.457‡
**Height**	9.0 cm	23	0.524‡	15	0.412‡	21	0.625‡
**Weight**	18 kg	5.0	0.140	6.1	0.245†	9.0	0.267‡
**Body mass index**	5.3 kg/m^2	−3.5	0.091	2.6	0.116	−1.5	0.043
**Body surface area**	0.21 m^2	14.4	0.286‡	9.1	0.328‡	14.8	0.442‡
**Lean body mass**	11 kg	11.2	0.251†	14.4	0.327‡	17.0	0.504‡
**Waist size**	14 cm	−1.3	0.034	1.9	0.082	2.0	0.060
**Hip size**	11 cm	5.9	0.138	4.7	0.210†	1.1	0.032
**Waist–hip ratio**	0.078	−7.7	0.206*	−4.7	0.167*	1.2	0.036
**Systolic BP**	14 mmHg	2.6	0.066	−4.4	0.193*	1.0	0.030
**Diastolic BP**	10 mmHg	−0.9	0.023	−5.5	0.238†	−1.9	0.056
**HbA1c**	9.0 mmol/mol	−4.7	0.128	−3.7	0.153	−4.0	0.116*
**Glucose**	1.8 mmol/L	−2.2	0.063	−3.5	0.144	−2.1	0.063
**Haematocrit**	3.4%	−5.5	0.110	−1.2	0.036	8.9	0.267‡
**Heart rate**	11 bpm	−15.5	0.450‡	−11.0	0.448‡	−14.1	0.422‡
**Overweight**	Yes	-12.7	0.178*	2.4	0.046	-6.8	0.099
**Hypertension**	Yes	5.9	0.073	−9.6	0.171*	−1.6	0.020
**Diabetes**	Yes	−19.0	0.136	−14.9	0.156*	−17.0	0.128*

ß is the change in dependent variable given a change of one standard deviation in the independent variable. R is the Pearson correlation. * P<0.05; † P<0.01; ‡ P<0.001. *BP* blood pressure.

Supplementary Table 8. Univariate demographic predictors of LV stroke volume.

		Males		Females		Both genders	
**Variable**	**Per**	*ß*	*r*	*ß*	*r*	*ß*	*r*
**Age**	6.8 years	−2.2	0.091	−1.3	0.090	−1.0	0.050
**Gender**	Female	N/A	N/A	N/A	N/A	−15.7	0.380‡
**Height**	9.0 cm	12.7	0.471‡	10.1	0.414‡	11.6	0.563‡
**Weight**	18 kg	2.01	0.089	4.9	0.298‡	5.2	0.251‡
**Body mass index**	5.3 kg/m^2	−2.9	0.120	2.5	0.168*	−0.5	0.026
**Body surface area**	0.21 m^2	5.4	0.228†	6.9	0.379‡	8.4	0.407‡
**Lean body mass**	11 kg	5.3	0.190*	10.7	0.374‡	9.1	0.439‡
**Waist size**	14 cm	−1.5	0.065	2.2	0.144	1.4	0.065
**Hip size**	11 cm	1.7	0.062	4.1	0.280‡	1.2	0.056
**Waist–hip ratio**	0.078	−4.0	0.169*	−2.3	0.127	0.6	0.029
**Systolic BP**	14 mmHg	2.5	0.105	−2.5	0.164*	0.9	0.043
**Diastolic BP**	10 mmHg	−0.8	0.034	−3.4	0.221†	−1.4	0.069
**HbA1c**	9.0 mmol/mol	−2.9	0.121	−2.8	0.176*	−2.8	0.131*
**Glucose**	1.8 mmol/L	−0.8	0.037	−3.2	0.197*	−1.6	0.079
**Haematocrit**	3.4%	−3.8	0.120	0.3	0.014	4.6	0.223‡
**Heart rate**	11 bpm	−9.3	0.426‡	−6.0	0.373‡	−8.1	0.392‡
**Overweight**	Yes	-7.8	0.172*	2.8	0.085	-3.3	0.077
**Hypertension**	Yes	4.2	0.083	−5.2	0.142	−0.4	0.008
**Diabetes**	Yes	−10.9	0.127	−10.6	0.171*	−10.9	0.132*

ß is the change in dependent variable given a change of one standard deviation in the independent variable. R is the Pearson correlation. * $P<0.05$; † $P<0.01$; ‡ $P<0.001$. *BP* blood pressure.

Supplementary Table 9. Univariate demographic predictors of LV ejection fraction.

		Males		Females		Both genders	
**Variable**	**Per**	*ß*	*r*	*ß*	*r*	*ß*	*r*
**Age**	6.8 years	0.68	0.137	0.41	0.097	0.46	0.099
**Gender**	Female	N/A	N/A	N/A	N/A	1.56	0.169†
**Height**	9.0 cm	−0.27	0.047	0.54	0.078	−0.50	0.109*
**Weight**	18 kg	−0.55	0.115	0.79	0.170*	−0.05	0.013
**Body mass index**	5.3 kg/m^2	−0.50	0.098	0.61	0.146	0.24	0.052
**Body surface area**	0.21 m^2	−0.53	0.106	0.92	0.180*	−0.21	0.045
**Lean body mass**	11 kg	−0.72	0.122	1.39	0.172*	−0.58	0.124*
**Waist size**	14 cm	−0.44	0.089	0.67	0.157*	0.12	0.027
**Hip size**	11 cm	−1.02	0.176*	0.83	0.205*	0.38	0.083
**Waist–hip ratio**	0.078	0.31	0.062	0.26	0.050	−0.10	0.021
**Systolic BP**	14 mmHg	0.59	0.116	0.24	0.058	0.24	0.054
**Diastolic BP**	10 mmHg	−0.127	0.025	0.027	0.006	−0.119	0.026
**HbA1c**	9.0 mmol/mol	-0.09	0.019	−0.41	0.091	−1.89	0.057
**Glucose**	1.8 mmol/L	0.22	0.048	−0.77	0.170*	−0.32	0.070
**Haematocrit**	3.4%	−0.47	0.072	0.55	0.092	−0.49	0.107
**Heart rate**	11 bpm	−0.11	0.023	0.36	0.079	0.17	0.038
**Overweight**	Yes	-0.03	0.003	0.95	0.102	0.55	0.058
**Hypertension**	Yes	0.54	0.051	0.60	0.058	0.54	0.051
**Diabetes**	Yes	-0.04	0.002	−1.36	0.077	−0.72	0.040

ß is the change in dependent variable given a change of one standard deviation in the independent variable. R is the Pearson correlation. * P<0.05; † P<0.01; ‡ P<0.001. *BP* blood pressure.

Supplementary Table 10. Univariate demographic predictors of RV end-diastolic volume.

		**Males**		**Females**		**Both genders**	
**Variable**	**Per**	*ß*	*r*	*ß*	*r*	*ß*	*r*
**Age**	6.8 years	−7.2	0.165*	−5.3	0.202*	−4.3	0.106
**Gender**	Female	N/A	N/A	N/A	N/A	−43	0.528‡
**Height**	9.0 cm	25	0.513‡	16	0.381‡	26	0.648‡
**Weight**	18 kg	6.2	0.151	6.9	0.242‡	11.3	0.282‡
**Body mass index**	5.3 kg/m^2	−3.4	0.078	3.2	0.123	−1.7	0.043
**Body surface area**	0.21 m^2	12.3	0.291‡	10.1	0.316‡	18.5	0.461‡
**Lean body mass**	11 kg	13	0.258†	16	0.321‡	22	0.550‡
**Waist size**	14 cm	−0.8	0.018	2.2	0.086	3.0	0.074
**Hip size**	11 cm	6.6	0.135	5.4	0.213†	0.2	0.006
**Waist–hip ratio**	0.078	−8.0	0.187*	−4.6	0.143	3.7	0.091
**Systolic BP**	14 mmHg	3.3	0.074	−6.9	0.266‡	0.8	0.020
**Diastolic BP**	10 mmHg	−2.4	0.056	−8.5	0.320‡	−3.6	0.089
**HbA1c**	9.0 mmol/mol	−4.0	0.094	−2.7	0.096	−3.0	0.074
**Glucose**	1.8 mmol/L	−3.1	0.074	−3.5	0.127	−2.2	0.055
**Haematocrit**	3.4%	−7.3	0.128	−0.4	0.012	12.8	0.318‡
**Heart rate**	11 bpm	−18.1	0.458‡	−13.3	0.477‡	−17.0	0.422‡
**Overweight**	Yes	-12.6	0.154	1.4	0.024	-7.9	0.095
**Hypertension**	Yes	4.1	0.045	−15.3	0.239†	−5.1	0.055
**Diabetes**	Yes	−19.5	0.122	−11.9	0.110	−15.8	0.099

ß is the change in dependent variable given a change of one standard deviation in the independent variable. R is the Pearson correlation. * P<0.05; † P<0.01; ‡ P<0.001. *BP* blood pressure.

Supplementary Table 11. Univariate demographic predictors of RV ejection fraction.

		Males		Females		Both genders	
**Variable**	**Per**	*ß*	*r*	*ß*	*r*	*ß*	*r*
**Age**	6.8 years	0.82	0.184*	0.59	0.142	0.54	0.117*
**Gender**	Female	N/A	N/A	N/A	N/A	3.5	0.375‡
**Height**	9.0 cm	0.05	0.011	0.88	0.128	−0.97	0.212‡
**Weight**	18 kg	−0.37	0.087	0.39	0.084	−0.40	0.086
**Body mass index**	5.3 kg/m^2	−0.38	0.085	0.18	0.043	0.13	0.029
**Body surface area**	0.21 m^2	−0.28	0.063	0.59	0.116	−0.65	0.142*
**Lean body mass**	11 kg	−0.45	0.086	0.80	0.099	−1.29	0.279‡
**Waist size**	14 cm	−0.33	0.074	0.21	0.052	−0.17	0.038
**Hip size**	11 cm	−0.59	0.116	0.40	0.100	0.50	0.109
**Waist–hip ratio**	0.078	0.20	0.046	−0.27	0.052	−0.77	0.165†
**Systolic BP**	14 mmHg	0.50	0.110	0.66	0.156*	0.29	0.062
**Diastolic BP**	10 mmHg	0.23	0.051	0.69	0.160*	0.30	0.065
**HbA1c**	9.0 mmol/mol	−0.29	0.067	−0.84	0.186*	−0.60	0.126*
**Glucose**	1.8 mmol/L	0.25	0.062	−0.79	0.176*	−0.36	0.078
**Haematocrit**	3.4%	0.16	0.028	0.16	0.027	−1.11	0.240‡
**Heart rate**	11 bpm	0.37	0.092	0.78	0.173*	0.68	0.148
**Overweight**	Yes	-0.24	0.028	0.63	0.068	0.39	0.041
**Hypertension**	Yes	1.34	0.142	1.85	0.179*	1.55	0.145*
**Diabetes**	Yes	−0.5	0.027	−3.0	0.172*	−1.8	0.097

ß is the change in dependent variable given a change of one standard deviation in the independent variable. R is the Pearson correlation. * $P<0.05$; † $P<0.01$; ‡ $P<0.001$. *BP* blood pressure.

Supplementary Table 12. Univariate demographic predictors of native T_1 values.

		Males		Females		Both genders	
**Variable**	**Per**	*ß*	*r*	*ß*	*r*	*ß*	*r*
**Age**	6.8 years	3.2	0.106	−2.5	0.072	−0.3	0.009
**Gender**	Female	N/A	N/A	N/A	N/A	4.8	0.072
**Height**	9.0 cm	7.7	0.219†	3.9	0.068	1.6	0.050
**Weight**	18 kg	9.6	0.326‡	5.2	0.134	6.3	0.188‡
**Body mass index**	5.3 kg/m²	7.5	0.235†	4.0	0.114	5.6	0.166*
**Body surface area**	0.21 m²	10.5	0.343‡	5.7	0.135	5.5	0.167*
**Lean body mass**	11 kg	12.4	0.339‡	10.1	0.150	3.4	0.104
**Waist size**	14 cm	8.5	0.273‡	2.0	0.058	4.4	0.132*
**Hip size**	11 cm	8.9	0.240†	4.8	0.141	6.3	0.187*
**Waist–hip ratio**	0.078	5.9	0.194*	−4.2	0.101	−0.2	0.007
**Systolic BP**	14 mmHg	1.07	0.034	−0.73	0.021	−0.37	0.012
**Diastolic BP**	10 mmHg	2.2	0.067	2.5	0.072	2.1	0.062
**HbA1c**	9.0 mmol/mol	1.40	0.049	0.95	0.026	1.13	0.034
**Glucose**	1.8 mmol/L	2.6	0.094	2.4	0.065	2.4	0.073
**Haematocrit**	3.4%	−0.5	0.012	−3.8	0.077	−2.8	0.086
**Heart rate**	11 bpm	4.5	0.160	2.3	0.061	3.5	0.106
**Overweight**	Yes	8.6	0.147	3.3	0.043	6.1	0.089
**Hypertension**	Yes	1.1	0.016	4.9	0.057	3.0	0.039
**Diabetes**	Yes	3.6	0.032	4.8	0.033	4.3	0.033

ß is the change in dependent variable given a change of one standard deviation in the independent variable. R is the Pearson correlation. * P<0.05; † P<0.01; ‡ P<0.001. *BP* blood pressure.

Supplementary Table 13. Univariate demographic predictors of extracellular volume.

		Males		Females		Both genders	
**Variable**	**Per**	*ß*	*r*	*ß*	*r*	*ß*	*r*
**Age**	6.8 years	0.25	0.136	−0.03	0.017	−0.01	0.006
**Gender**	Female	N/A	N/A	N/A	N/A	2.7	0.571‡
**Height**	9.0 cm	0.06	0.029	0.40	0.118	−0.83	0.348‡
**Weight**	18 kg	−0.35	0.197*	−0.37	0.164*	−0.67	0.281‡
**Body mass index**	5.3 kg/m^2	−0.44	0.227†	−0.40	0.194*	−0.26	0.108
**Body surface area**	0.21 m^2	−0.29	0.155	−0.31	0.124	−0.85	0.356‡
**Lean body mass**	11 kg	−0.36	0.163	−0.52	0.131	−1.20	0.502‡
**Waist size**	14 cm	−0.43	0.225†	−0.38	0.186*	−0.54	0.222‡
**Hip size**	11 cm	−0.63	0.280‡	−0.33	0.171*	−0.04	0.015
**Waist–hip ratio**	0.078	−0.10	0.051	−0.38	0.154	−0.79	0.330‡
**Systolic BP**	14 mmHg	−0.32	0.168*	−0.59	0.295‡	−0.71	0.296‡
**Diastolic BP**	10 mmHg	−0.12	0.064	−0.47	0.228‡	−0.48	0.197‡
**HbA1c**	9.0 mmol/mol	0.34	0.201*	−0.11	0.052	0.09	0.037
**Glucose**	1.8 mmol/L	0.22	0.120	−0.10	0.045	−0.02	0.009
**Haematocrit**	3.4%	−0.91	0.379‡	−1.21	0.434‡	−1.50	0.632‡
**Heart rate**	11 bpm	−0.19	0.114	−0.29	0.136	−0.16	0.067
**Overweight**	Yes	-0.72	0.203*	-0.71	0.161*	-0.56	0.115*
**Hypertension**	Yes	0.13	0.032	−0.74	0.150	−0.37	0.068
**Diabetes**	Yes	1.68	0.247*	−0.60	0.072	0.05	0.053

ß is the change in dependent variable given a change of one standard deviation in the independent variable. R is the Pearson correlation. * $P<0.05$; † $P<0.01$; ‡ $P<0.001$. *BP* blood pressure.

Supplementary Table 14. Univariate demographic predictors of T_2 values.

		Males		Females		Both genders	
**Variable**	**Per**	*ß*	*r*	*ß*	*r*	*ß*	*r*
**Age**	6.8 years	−0.26	0.133	−0.18	0.090	−0.25	0.125*
**Gender**	Female	N/A	N/A	N/A	N/A	1.16	0.288‡
**Height**	9.0 cm	0.05	0.020	0.31	0.099	−0.32	0.156†
**Weight**	18 kg	−0.42	0.241†	−0.39	0.183*	−0.51	0.258‡
**Body mass index**	5.3 kg/m^2	−0.51	0.266†	−0.42	0.219†	−0.38	0.189‡
**Body surface area**	0.21 m^2	−0.38	0.204*	−0.35	0.146	−0.55	0.271‡
**Lean body mass**	11 kg	−0.44	0.198*	−0.58	0.155	−0.65	0.323‡
**Waist size**	14 cm	−0.54	0.284‡	−0.31	0.155	−0.44	0.219‡
**Hip size**	11 cm	−0.38	0.179*	−0.28	0.144	−0.14	0.072
**Waist–hip ratio**	0.078	−0.60	0.312‡	−0.28	0.115	−0.61	0.301‡
**Systolic BP**	14 mmHg	−0.50	0.261†	−0.78	0.405‡	−0.73	0.370‡
**Diastolic BP**	10 mmHg	−0.18	0.091	−0.80	0.406‡	−0.60	0.294‡
**HbA1c**	9.0 mmol/mol	−0.41	0.224†	−0.44	0.215†	−0.42	0.212‡
**Glucose**	1.8 mmol/L	−0.33	0.197*	−0.41	0.201*	−0.40	0.206‡
**Haematocrit**	3.4%	−0.32	0.128	−0.75	0.277†	−0.69	0.345‡
**Heart rate**	11 bpm	−0.97	0.555‡	−1.31	0.642‡	−1.10	0.554‡
**Overweight**	Yes	-0.99	0.273†	-1.00	0.228†	-0.90	0.214‡
**Hypertension**	Yes	−0.76	0.188*	−1.20	0.255†	−0.99	0.217‡
**Diabetes**	Yes	−1.78	0.246†	−1.92	0.234†	−1.83	0.227‡

ß is the change in dependent variable given a change of one standard deviation in the independent variable. R is the Pearson correlation. * P<0.05; † P<0.01; ‡ P<0.001. *BP* blood pressure.

Supplementary Table 15. Pearson correlations between independent variables.

	Age	Gender	Height	BMI	WHR	SBP	DBP	HbA1c	HR
**Age**		-0.081	0.065	0.265	0.341	0.259	0.400	0.262	0.129
**Gender**	-0.081		-0.683	0.102	-0.431	-0.166	-0.102	-0.022	0.061
**Height**	0.065	-0.683		-0.150	0.267	0.148	0.092	-0.078	-0.109
**BMI**	0.265	0.102	-0.150		0.483	0.149	0.171	0.253	0.228
**WHR**	0.341	-0.431	0.267	0.483		0.263	0.232	0.340	0.213
**SBP**	0.259	-0.166	0.148	0.149	0.263		0.752	0.157	0.244
**DBP**	0.400	-0.102	0.092	0.171	0.232	0.752		0.183	0.234
**HbA1c**	0.262	-0.022	-0.078	0.253	0.340	0.157	0.183		0.220
**HR**	0.129	0.061	-0.109	0.228	0.213	0.244	0.234	0.220	

BMI body mass index, *WHR* waist-hip ratio, *SBP* systolic blood pressure, *DBP* diastolic blood pressure, *HR* heart rate

Supplementary Table 16. Variance inflation factors (VIF) of independent variables.

**Independent variable**	**VIF**
**Age**	1.3
**Gender**	2.3
**Length**	2.0
**Body mass index**	1.5
**Waist-hip ratio**	2.1
**Systolic blood pressure**	2.4
**Diastolic blood pressure**	2.6
**HbA1c**	1.2
**Heart rate**	1.2
